# Reduced GeO_2_ Nanoparticles: Electronic Structure of a Nominal GeO_x_ Complex and Its Stability under H_2_ Annealing

**DOI:** 10.1038/srep17779

**Published:** 2015-12-04

**Authors:** Jia Zhao, Linju Yang, John A. McLeod, Lijia Liu

**Affiliations:** 1 Jiangsu Key Laboratory for Carbon-Based Functional Materials & Devices, Institute of Functional Nano and Soft Materials (FUNSOM), Soochow University-Western University Center for Synchrotron Radiation Research, Soochow University, Suzhou, Jiangsu, 215123 China

## Abstract

A nominal GeO_x_ (x ≤ 2) compound contains mixtures of Ge, Ge suboxides, and GeO_2_, but the detailed composition and crystallinity could vary from material to material. In this study, we synthesize GeO_x_ nanoparticles by chemical reduction of GeO_2_, and comparatively investigate the freshly prepared sample and the sample exposed to ambient conditions. Although both compounds are nominally GeO_x_, they exhibit different X-ray diffraction patterns. X-ray absorption fine structure (XAFS) is utilized to analyse the detailed structure of GeO_x_. We find that the two initial GeO_x_ compounds have entirely different compositions: the fresh GeO_x_ contains large amorphous Ge clusters connected by GeO_x_, while after air exposure; the Ge clusters are replaced by a GeO_2_-GeO_x_ composite. In addition, the two GeO_x_ products undergo different structural rearrangement under H_2_ annealing, producing different intermediate phases before ultimately turning into metallic Ge. In the fresh GeO_x_, the amorphous Ge remains stable, with the GeO_x_ being gradually reduced to Ge, leading to a final structure of crystalline Ge grains connected by GeO_x_. The air-exposed GeO_x_ on the other hand, undergoes a GeO_2_→GeO_x_→Ge transition, in which H_2_ induces the creation of oxygen vacancies at intermediate stage. A complete removal of oxides occurs at high temperature.

Ge is one of the promising candidates for anode materials in Li-ion batteries^1^,^2^. It has a theoretical capacity as high as 1600 mA h g^−1^ (upon formation of a Li_4.4_Ge alloy) and excellent Li^+^ diffusion rate at room temperature^1^. However, the drastic volume expansion in crystalline Ge that occurs after Li insertion leads to capacity fading which limits its use in practical Li-ion devices^3^. Researchers have been seeking methods to enhance the stability of Ge anodes, such as minimizing the size of Ge^4^,^5^, surface functionalization^2^,^6^, morphology engineering^7^,^8^ and forming a composite structure by coating Ge with a layer of carbon^9^,^10^. Recently, amorphous GeO_x_ (x < 2) structures have attracted great interest due to their ability to enhance the cycling life of Li-ion batteries^11^,^12^,^13^,^14^. Compared to crystalline Ge, oxidized Ge is lower cost, has better chemical stability, and improved cyclability. In fact, it has been reported that GeO_2_ is able to deliver a capacity up to 2152 mA h g^−1^ if it reversibly stores 8.4 Li^+^ (reactions (1) and (2))^14^,^15^,^16^.


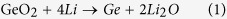



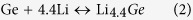


It has been proposed that presence of Ge^0^ in GeO_2_ has a unique role in that it can serve as a catalyst to drive reaction (1) in the inverse direction, hence the formation of LiO_2_ is reversible^17^. This catalytic effect of Ge has been demonstrated by Seng *et al.* using a GeO_2_/Ge/C nanocomposite as an anode for a Li battery test^17^. A nominal GeO_x_ structure contains a mixture of Ge dioxides and sub-oxides, as well as elemental Ge. It is critical to understand the composition and the structure of GeO_x_ to achieve better control of the crystallinity and grain size of the different constituents in terms of improving the battery performance.

Although there has been a large amount of work on synthesizing nanostructured GeO_x_ with new configurations^12^,^14^,^18^, less attention has been given to understanding the starting material itself before introducing it into a battery test. GeO_x_ contains a mixture of Ge, GeO_x_, and GeO_2_, and in an oxidizing (or reducing) environment, these three components can transform from one species to another^19^,^20^,^21^. In fact, GeO_x_ has been deliberately synthesized and served either as the precursor for making Ge nanocrystals and/or GeO_2_ nanostructures for the purpose of fabricating electronic and optical devices^22^,^23^,^24^. Although there have been relatively large amount of early studies done on Ge nanocrystal embedded GeO_x_ or SiO_x_ thin films for the purpose of electronic devices fabrication, few reports are available on the structure characterization of the free-standing Ge/GeO_x_/GeO_2_ nanocomposite in terms of battery applications. In particular, since amorphous Ge and GeO_x_ are more promising for Li-ion anodes than their crystalline counterparts, the standard crystal structure analysis tool X-ray diffraction (XRD) is no longer capable of characterizing the crystal structure of GeO_x_. Transmission electron microscopy (TEM) with high resolution can tell the crystallinity of individual nanoparticles. However, for amorphous structure, distinguishing different components (e.g. Ge and GeO_x_) almost entirely relies on the contrast of the image. In addition, the small sampling size of TEM lacks information on the averaged chemical composition of the material.

Aside from the crystal structure, understanding the chemical components in GeO_x_ from the electronic structure perspective is also important. GeO_x_ contains Ge in various oxidation states, from Ge^0^ up to Ge^4+^. The conventional characterization technique for studying the electronic structure of GeO_2_/Ge materials is photoelectron spectroscopy^21^. This technique requires the samples to have a clean surface and be in good electrical contact with the substrate. However, nanostructured GeO_x_ for Li-ion battery applications are more often in the form of powder. The GeO_x_ therefore have irregular surfaces, which makes removing surface impurities and ensuring good electrical contact very difficult. Consequently, characterizing the electronic structure of these materials with photoelectron spectroscopy is very challenging. X-ray absorption spectroscopy, on the other hand, is an alternative tool for electronic structure characterization that is flexible in terms of sample preparation. X-ray absorption fine structure (XAFS) probes the local environment of an element of interest in the material. The spectrum originates from the interference between incoming and outgoing electrons after single and/or multiple scattering. As a result, it is a local structure probe and doesn’t require the sample to be crystalline. By measuring the Ge K-edge XAFS, the chemical environment of Ge, such as its oxidation state (from the X-ray absorption near-edge structure, XANES), the coordination number, and the bond distance between Ge and the nearest neighbors (from the extended X-ray absorption fine structure, EXAFS) can be obtained^14^,^25^,^26^,^27^.

GeO_x_ nanostructures can be synthesized using various approaches, such as hydrothermal methods^13^, hydrolysis^17^, and by chemical reduction^2^,^11^. Herein we study the electronic structure of GeO_x_ nanoparticles synthesized by simple chemical reduction. Such method produces GeO_x_ which contains GeO_2_, Ge sub-oxides, and most interestingly, Ge^11^. This serves as a good starting point to investigate the electronic structure of the GeO_x_, and the transitions between the three components under various conditions. We first compare the freshly prepared GeO_x_, and the one stored in ambient condition for 3 days. These two samples were then used as starting materials, and were annealed in H_2_ at various temperatures. The change in the chemical environment of Ge under different annealing temperatures is studied.

## Results and Discussion

**Figure 1:**
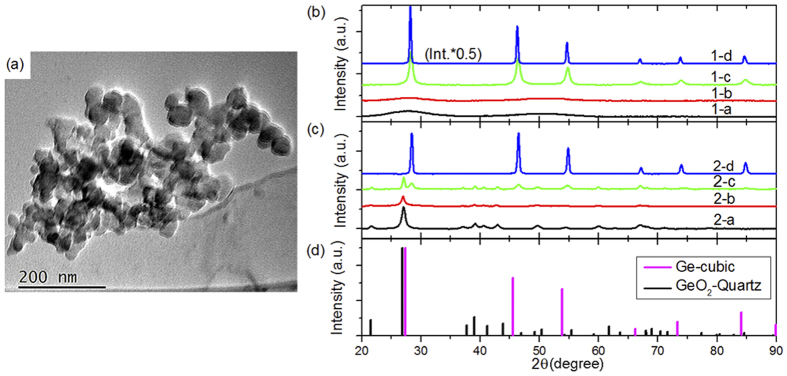
(**a**) TEM image of as-prepared fresh GeO_x_ nanoparticles. (**b**–**d**) XRD patterns of GeO_x_ nanoparticles before and after annealing in comparison with standards. (**b**) fresh GeO_x_ (sample series 1); (**c**) air-exposed GeO_x_ (sample series 2); (**d**) standard XRD patterns of cubic Ge and quartz GeO_2_^33^,^34^. Labels in (**b**) and (**c**): (**a**) as-prepared samples; (**b**) samples annealed at 300 °C; (**c**) samples annealed at 500 °C; (**d**) samples annealed at 700 °C. The intensity of 1-d is reduced in half to fit the panel.

The freshly prepared GeO_x_ nanoparticles have an average size of 50 nm. Figure 1a shows a representative transmission electron microscopy (TEM) image of these particles. X-ray diffraction (XRD) measurement reveals that the fresh GeO_x_ and air-exposed GeO_x_ have very different crystal structure (Fig. 1b–d). The fresh GeO_x_ only contains two broad peaks, indicating its amorphous nature. The air-exposed GeO_x_, on the other hand, shows well-resolved features that resemble crystalline GeO_2_ with a quartz structure. After annealing in H_2_ at 300 °C, the XRD patterns of both fresh GeO_x_ and air-exposed GeO_x_ are both broadened to some extent, however, the features characteristic of GeO_2_ are still present in the latter. When the annealing temperature is increased to 500 °C, fresh GeO_x_ crystallizes into an fcc structure, with diffraction peaks matching cubic-phase metallic Ge. These features are further sharpened under annealing at 700 °C. As for air-exposed GeO_x_, the 500 °C annealing results in a mixed phase containing both GeO_2_ and Ge. From the intensity of the two peak series, the GeO_2_ is still the major component. A full conversion from GeO_2_ to Ge is observed at 700 °C annealing.

We analyse the crystalline grain size of our samples using the Scherrer equation (Eq. 3),


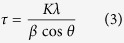


in which τ is the crystalline size, K is the shape factor, λ is the wavelength of the incident X-ray, β is the full width at half maximum. In this case, the values of β for 700 °C annealed fresh GeO_x_ and air-exposed GeO_x_ are 0.0055 rad (0.318) and 0.0065 rad (0.370), respectively. Taking K=0.89 (the typical value of the shape factor) and λ = 1.5406 Å, we can obtain the averaged crystalline sizes for annealed fresh GeO_x_ and air-exposed GeO_x_ are 26 nm and 22 nm, respectively. The crystalline domain size of metallic Ge by annealing fresh GeO_x_ is slightly larger than that of air-exposed GeO_x_.

Although after the final annealing, the two GeO_x_ possess very similar XRD patterns, the initial and intermediate phases are significantly different. Since XRD is only sensitive to structure of long-range order (i.e. crystalline), we then turn to XAFS to further examine the local structure of GeO_x_ nanoparticles.

**Figure 2:**
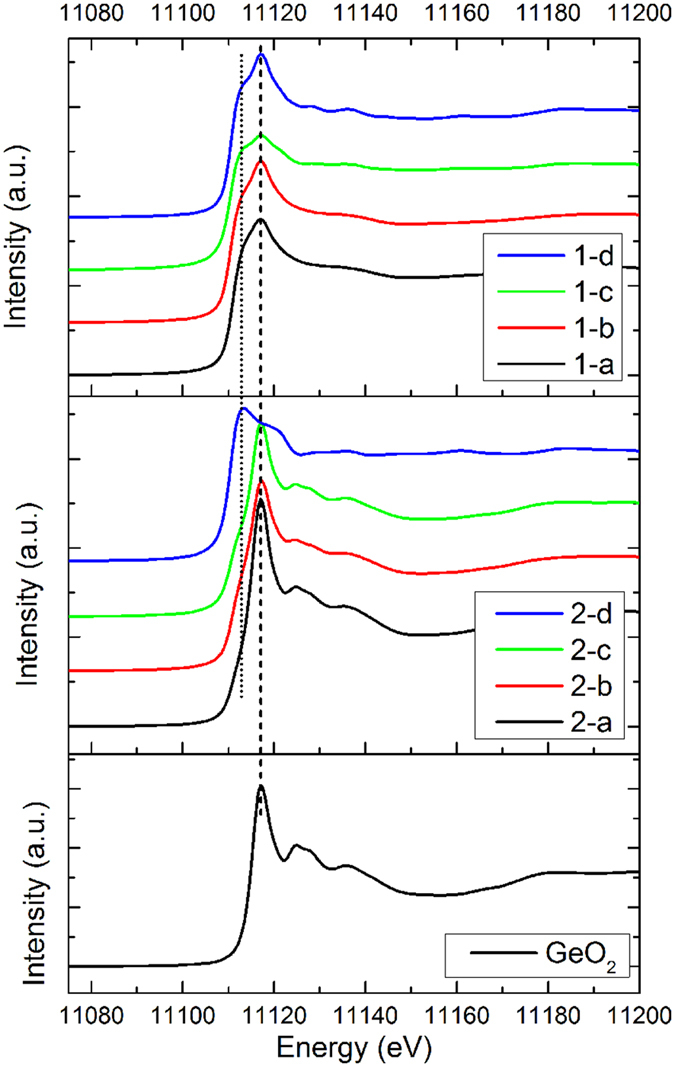
Ge K-edge XANES of GeO_x_ nanoparticles. sample series 1: fresh GeO_x_, series 2: air-exposed GeO_x_; (**a**) as-prepared samples, (**b**) samples annealed at 300 °C, (**c**) samples annealed at 500 °C, (**d**) samples annealed at 700 °C.

Figure 2 shows the Ge K-edge XANES for all GeO_x_ samples, before and after annealing, in comparison with commercial GeO_2_ powder. As a fully oxidized Ge standard, the XANES of GeO_2_ is dominated by one sharp peak (marked by the dashed line in Fig. 2), which corresponds to the transition of an electron from the Ge 1s core level to an unoccupied 4p state. Compared to GeO_2_, both GeO_x_ samples, with or without heat treatment, have additional features at lower energy (marked by the dotted line in Fig. 2), indicating the presence of Ge with an oxidation states lower than that in GeO_2_. Recall from the XRD pattern in Fig. 1 that the fresh GeO_x_ is amorphous until annealed at a temperature above 500 °C. From XANES, we can see that there are both Ge- and GeO_2_-like structures in the fresh GeO_x_, and the lack of diffraction peaks in the XRD suggests that they are amorphous. Under annealing at 500 °C and 700 °C, crystalline Ge appears (as confirmed by XRD, refer back to Fig. 1), although there is still amorphous GeO_2_ present as evidenced by XANES. On the other hand, air-exposed GeO_x_ is dominated by GeO_2_. A slight broadening at the lower energy side of the main peak is caused by the presence of Ge sub-oxides and Ge. A sudden transition occurs after annealing at 700 °C, in which almost all the GeO_2_ disappears, and the spectrum is dominated by a low-valent species Ge (spectrum 2-d). The energy onset and the spectral profile resembles the previously reported metallic Ge^2^,^28^. Unlike the fresh GeO_x_ which has a mixture of metallic Ge and amorphous GeO_2_, air-exposed GeO_x_ after 700 °C turns almost completely to metallic Ge. We note that the XRD patterns for the fresh and air-exposed samples after annealing at 700 °C are essentially the same, but the metallic Ge-related feature in the XANES is more intense for the air-exposed sample than the fresh sample, suggesting that in the air exposed sample, a larger fraction of the available Ge is converted to cubic Ge than in the fresh sample. This is further corroborated by noting that the air-exposed sample lacks a clear GeO_2_-related feature in the XANES; suggesting that so much Ge has been converted to metallic Ge that a distinct GeO_2_ phase is unable to form. Recall that the air-exposed sample annealed at 700 °C exhibits a smaller grain size for metallic Ge than the fresh sample annealed at 700 °C. If any Ge oxide remaining in these samples after annealing at 700 °C occupies the space between the grains, if these grains are close-packed then the smaller grains in the air-exposed sample will lead to smaller domains of Ge oxide; possibly too small for a distinct GeO_2_ lattice to form.

**Figure 3:**
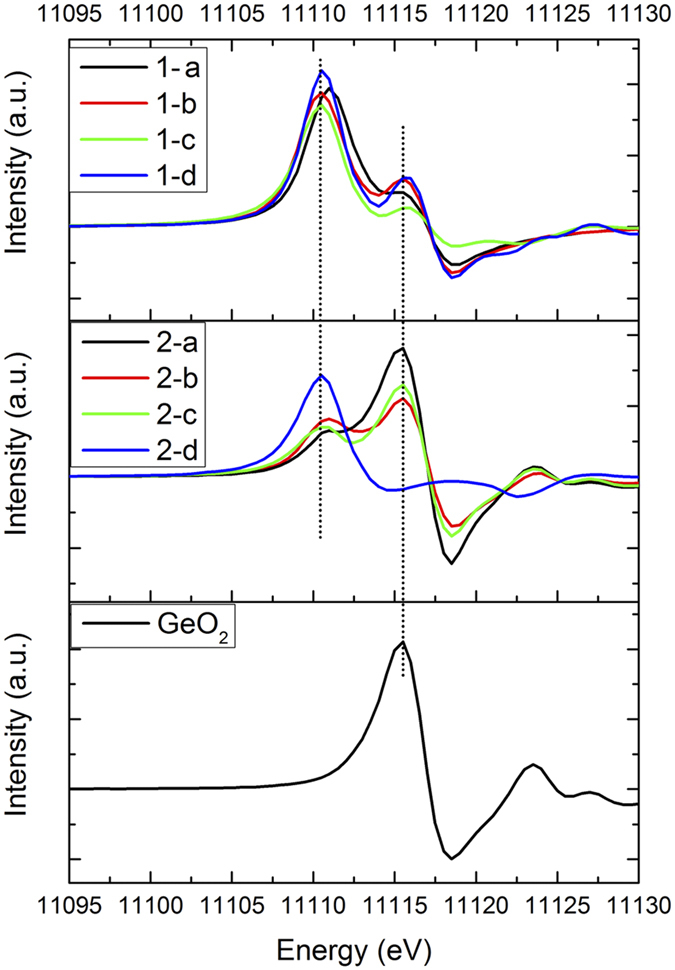
Ge K-edge XANES of GeO_x_ nanoparticles plotted in 1^st^ derivatives. The legend is the same as the one shown in Fig. 2.

A closer examination of the oxidation states in Ge can be performed by examining the 1^st^ derivative of the XANES spectra. The plots are shown in Fig. 3, and the peak positions (the edge jump) correspond to the oxidation states of Ge in the samples. This method allows us to obtain the evolution of Ge oxidation states during annealing in detail. The lower energy dashed line marks the edge jump of Ge^0^ and the higher energy line marks the one of Ge^4+^. Interestingly, the evolution of the edge jump positions as a function of annealing temperature is quite different between the two sets of GeO_x_.

For fresh GeO_x_ at room temperature (spectrum 1-a in Fig. 3), the edge jump is at energy slightly higher than Ge^0^, while after annealing, all the edge jumps are aligned to the position of Ge^0^ regardless of temperature. Instead of Ge^0^, the Ge in fresh GeO_x_ are slightly oxidized (i.e. 0 < x ≤ 2). After annealing at 300 °C, GeO_x_ undergoes a disproportionation reaction, producing Ge and GeO_2_ (reaction (4)), leading to the lower-energy shift of the main peak position, and a better-defined GeO_2_ edge jump.


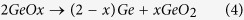


The disproportionation reaction of GeO_x_ into Ge and GeO_2_ is commonly observed in Ge nanostructure synthesis. The reaction takes place when GeO_x_ is annealed in a non-oxidizing environment, and is considered as one of the intermediate stages in forming a Ge-GeO_2_ complex^22^. It has been observed experimentally using XANES that annealing GeO_x_ thin films under N_2_ results in a low-energy shift in the shoulder feature and an intensity increase in GeO_2_ feature^27^,^29^. At 500 °C annealing, the GeO_2_ decreases noticeably, while Ge remains almost unchanged. At this temperature, the reduction of GeO_x_ starts to take place and O is being removed out of the system. The amorphous Ge clusters starts to crystalize. At 700 °C, phase separation takes place, Ge forms large crystalline grains and the left over GeO_2_ migrates to the grain boundary.

As for air-exposed GeO_x_, the one at room temperature contains both fully oxidized GeO_2_ and Ge sub-oxide. The Ge sub-oxide component exhibit a progressive shift at alleviated annealing temperature until it becomes Ge^0^, while the edge jump of the GeO_2_ component remains stable but the intensity decrease until it totally disappears as the temperature reaches 700 °C.

The results above demonstrate that the fresh GeO_x_ and air-exposed GeO_x_ has distinct compositions, and because of this, they undergo different crystallization processes under H_2_ annealing, even though the ultimate products have identical XRD patterns. The fresh GeO_x_ is always a mixture of Ge and GeO_2_, while air-exposed GeO_x_ is dominated by GeO_2_ at low and intermediate temperatures with a gradual emergence of Ge, and GeO_2_ is fully converted to Ge^0^ at high annealing temperature.

**Figure 4:**
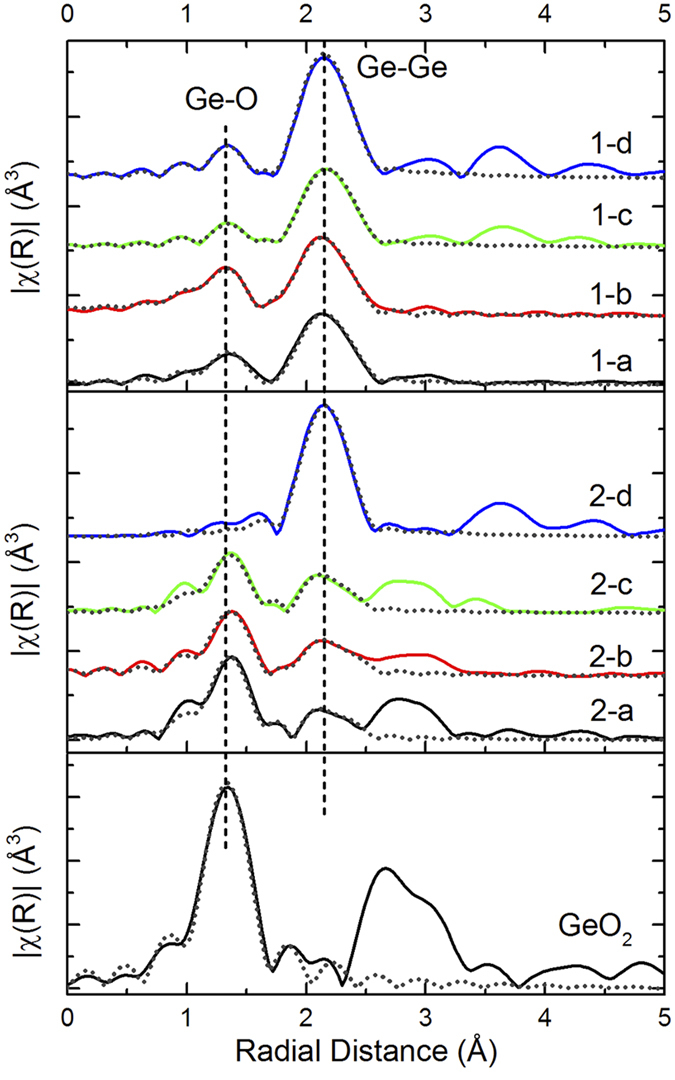
Fourier transforms of Ge K-edge *k*^3^-weighted EXAFS data in R-space with first-shell fits. Solid lines: experimental spectra; dotted lines: fitted spectra. Sample series 1: fresh GeO_x_, series 2: air-exposed GeO_x_. The labels are the same as the ones in Fig. 2.

The Fourier transformed EXAFS spectra of GeO_x_ are shown in Fig. 4. The amplitude was fitted using the IFFEFIT package at the R-range between 1.0 Å and 2.5Å (first shell). Detailed structural parameters, such as the Ge coordination number (CN), Ge-O and Ge-Ge bond distance (R), Debye Waller factor (σ^2^), and inner potential shift (ΔE) are listed in Table 1.

It can be clearly seen from Fig. 4 that the fresh GeO_x_ contains significant amount of Ge-Ge bonding, with bond distance of ~2.45Å. This bond length is consistent with previous EXAFS studies of Ge-Ge bonding^30^. The CN_Ge-Ge_ is less than the one of metallic Ge, indicating its amorphous nature. As for the oxide component, Ge-O bonds are present in the material with bond lengths close to that of GeO_2_. Compared to GeO_2_, only the first shell Ge-O is present in GeO_x_; the features from second shell (features around 2.5Å to 3.2Å) are missing. The low CN_Ge-O_ means GeO_x_ are highly under-coordinated compared to GeO_2_.This further confirms that the GeO_x_ components in the fresh samples lack long range order. The as-prepared GeO_x_ contains both GeO_x_ and Ge clusters in an amorphous form.

Under annealing at 300 °C, the CN_Ge-O_ remains almost unchanged (within the error range), while the CN_Ge-Ge_ increases. The values deduced from the fitting further supports our proposed reaction of GeO_x_ disproportionation at 300 °C (reaction 4). During the reaction, O atom is transferred from one Ge to another Ge, leaving one Ge with dangling bond, and then two adjacent Ge joined together to form Ge-Ge bond. A similar reaction scheme was proposed by Wang *et al.* on vacuum annealed GeO film^21^, and our result is consistent with their observation.

The Ge-O bond almost disappears after annealing at 500 °C, while there is a slight decrease in CN_Ge-Ge_. At this stage, reduction of GeO_x_ (reaction (5)) becomes the dominant reaction in the system. This reaction leads to the consumption of GeO_x_, hence a significant decrease of CN_Ge-O_.


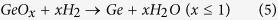


As the temperature further increases, thermal induced crystallization takes place, the Ge clusters becomes highly coordinated, with CN_Ge-Ge_ approaches metallic Ge.

We should note that CN_Ge-Ge_, CN_Ge-O_, and the intensity of the XANES features are smaller for the sample annealed at 500 °C than for the other samples (refer back to Fig. 2 and Table 1). There are two possible explanations for this: One is that at 500 °C disproportionation into cubic Ge and amorphous GeO_2_ is complete, but these phases exist as finely mixed nanoscale domains (this is supported by the rather broad XRD pattern of cubic Ge from the sample annealed at 500 °C, refer back to Fig. 1(b)). These very small domains indicate a relatively large amorphous boundary region, in which the general lack of structure would lead to a more intense background in the XAFS spectrum that reduces the relative amplitude of the fine structure features related to the Ge and GeO_2_ and phases. After annealing at 700 °C the respective cubic Ge and amorphous GeO_2_ domains are large enough to be resolved separated in the XAFS spectrum. On the other hand, it is possible that the pellet prepared from the 500 °C sample was slightly too thick for XAFS and consequently the XANES features and coordination numbers are reduced by the “thickness effect”^31^. We would like to stress that the ratio of CN_Ge-O_ to CN_Ge-Ge_ is the same for the samples annealed at 500 °C and 700 °C (~0.3), and both are lower than those for the samples annealed at 300 °C (0.46) and as-prepared (0.65). The thickness of the XAFS sample would affect CN_Ge-O_ and CN_Ge-Ge_ equally, so this observation is independent of possible measurement artefacts. Therefore regardless of whether the anomalous aspects of the XANES and EXAFS from the sample annealed at 500 °C are due to nanoscale GeO_2_, Ge phase separation, or are due to a measurement artefact, the discussion above shows that the disproportionation reaction (4) is largely complete after annealing at 500 °C, and that higher temperatures simply improve phase separation and growth of larger crystals of Ge. We should also point out that a previous report shows that high temperature (above 500 °C) annealing induces the crystallization of GeO_x_^23^, which would lead to O migration to the edges of the cubic Ge domains. This could also lead to the increase in the CN_Ge-O_ from the sample annealed at 500 °C to the one annealed at 700 °C.

As for the air-exposed GeO_x_, the material retains GeO_2_-like features from room temperature up to 500 °C. The room temperature sample contains mostly undercoordinated GeO_2_, and the CN_Ge-O_ is much closer to GeO_2_ compared to fresh GeO_x_. The second shell component of radial distance above 2.4Å is also present, so that air-exposed GeO_x_ is more of a GeO_2_-like crystalline structure. This also explains why GeO_2_-like pattern only appears in the XRD of air-exposed GeO_x_ but not the fresh ones. Small amounts of Ge-Ge bonds are present in the sample too, but from their low CN and long Ge-Ge distance, they are unlikely to be Ge clusters. Instead, the system is more like GeO_2_ with oxygen vacancies, in which Ge is present as dangling bonds.

Unlike fresh GeO_x_, the disproportionation reaction does not happen in the air-exposed GeO_x_ sample system. From Table 1, we see a decrease in CN_Ge-O_, while CN_Ge-Ge_ remains almost constant at 300 °C. Annealing the GeO_x_ in a reduced environment only partially removes O atoms (i.e. amorphorization), following the reaction (6), which produces a highly oxygen-deficient GeO_2_ structure. Meanwhile, the Ge-Ge distance gets shorter, indicating the formation of amorphous Ge cluster.


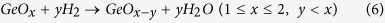


At 500 °C, the decrease of CN_Ge-O_ slows down. Recall from Fig. 1, metallic Ge features start to appear in the XRD, so at this stage, structural rearrangement start to occur. Note the Ge-O long-range order also improves as seen in Fig. 4. A system with many oxygen vacancies is not thermodynamically stable, so O migrates, and produces GeO_2_ and adjacent Ge start to join each other forming Ge-Ge bonds. At 700 °C, the high temperature leads to a total reduction of GeO_x_ (reaction (7)), leading to a sudden transition from GeO_x_ to Ge. Upon the removal of almost all O in GeO_x_, the Ge grows into cubic crystallites. The relative rapidity of this transition may explain why these crystallites are somewhat smaller than those that form during the more gradual crystallization during annealing in the fresh GeO_x_ samples. As the O is almost all removed, a distinct GeO_x_ phase no longer exists. This is evidenced by the lack of a clear GeO2-related feature in the XANES spectrum from the air-exposed sample annealed at 700 °C (refer back to Fig. 2), as well as the anomalously short Ge-O bond length obtained from EXAFS fitting (see Table 1). The remaining O is likely only found as a capping layer on the Ge crystallites.


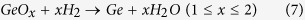


**Figure 5:**
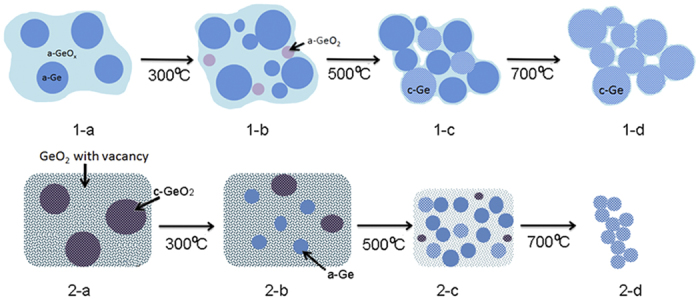
Illustration of the structure of GeO_x_ nanoparticles, fresh (1-a) and air-exposed (2-a), and their compositions under H_2_ annealing. The labels are the same as the ones in Fig. 2.

The structures of the two GeO_x_ samples and their behaviour under H_2_ annealing can be summarized in the scheme shown in Fig. 5. The fresh GeO_x_ contains both elemental Ge and GeO_x_ (x closes to 1), both are amorphous and present in comparable amounts. H_2_ annealing induces the disproportionation and reduction of GeO_x_, forming more Ge clusters. Ge clusters crystalize at high annealing temperatures, and grain boundaries are filled with GeO_x_. Air-exposed GeO_x_, on the other hand, can be modelled as GeO_2_ crystallites with O vacancies. Ambient air introduces oxygen, and the initial product is mostly crystalline GeO_2_ with GeO_x_ where x closes to 2. H_2_ annealing gradually creates more oxygen vacancies, reducing the presence of crystalline GeO_2_ and leading to the formation of more Ge dangling bonds. The GeO_x_ structure is completely eliminated at the temperature of 700 °C, when a transition to crystalline Ge occurs due to the H_2_ reduction reaction.

## Conclusion

As nominal GeO_x_ compounds, the actual compositions could vary significantly from material to material, depending on the synthesis strategies and post-treatment conditions. We have demonstrated that the exact composition and structure of GeO_x_ and its structural evolution during annealing can be successfully analyzed using XAFS. In our model system, GeO_x_ nanoparticles were synthesized by chemical reduction of GeO_2_, freshly prepared GeO_x_ is found consisting of Ge clusters and GeO_x_ (x closes to 1), both in amorphous forms. Such GeO_x_ undergoes disproportionation when annealed in a reducing environment. The amount of amorphous Ge increases, and finally crystallizes into metallic Ge, with GeO_x_ presents at the grain boundaries. However, if the GeO_x_ is exposed to ambient conditions, the elemental Ge domains are quickly replaced by oxides, and the nanoparticles turn into a GeO_2_-like structure. Once such structure formed, the GeO_x_ (x closes to 2) can be slowly reduced by H_2_, under mild temperature, producing small Ge crystallites embedded in GeO_x_ matrix. Once the temperature reaches 700 °C, there is a complete conversion of GeO_x_ to Ge. The composition of GeO_x_ can change significantly from its initial composition after exposure to oxygen, and this exposure also affects how the structural rearrangement takes place during post-treatment. These findings are of paramount importance to developing GeO_x_ anodes for Li-ion batteries. In particular, they highlight the importance of carefully controlled synthesis of GeO_x_ anodes – as even under identical preparation conditions, the composition and crystal structure can completely change based on whether or not the samples are exposed to ambient conditions. A controlled regime of air exposure followed by annealing in H_2_ can further tailor the composition of the material. All the samples investigated here have nominal GeO_x_ structures, but each of them possesses unique structures, which are identified by XAFS. With this in mind, the next step of the research includes the examination of Li battery performance with integration of these materials. The device performance can then be related to the fundamental structures of the GeO_x_. Amorphous Ge is preferable to crystalline Ge for Li-storage, but smaller clusters are also preferable to larger clusters. If the composition of the GeO_x_ is tunable, we will be able to find which configuration is desired as an anode material.

## Methods

### Material Synthesis

GeO_x_ nanoparticles were prepared using a chemical reduction method^11^,^24^. 2.0 g of GeO_2_ (99.99%, Aladdin) was first dissolved in 36 mL deionized water, and 7 mL of NH_4_OH (28%-30% NH_3_, Aladdin) was added. Freshly prepared NaBH_4_ (98%, Aladdin) solution (3.616 g in 20 mL deionized water) was quickly added to the mixture. The solution was vigorously stirred for 20h at room temperature. The resulting product was then filtered, washed with deionized water, and dried under vacuum at 50 °C. The freshly prepared GeO_x_ was divided into two parts, one sealed in a glass vial and kept in a glove box filled with N_2_ (denoted as fresh GeO_x_), and the other one was kept in a desiccator (humidity <3%) for 3 days (denoted as air-exposed GeO_x_). H_2_ annealing was conducted in a tube furnace. GeO_x_ was placed in a combustion crucible at the centre of the tube, and 2% H_2_ was introduced to the tube at a rate of 50 sccm (standard-state cubic centimetre per minute). The samples were annealed at 300 °C, 500 °C and 700 °C, respectively, under the H_2_ flow. The temperature was increased at a rate of 2.1 °C/min, and held at the desired temperature for 30 min. After annealing, the samples were cooled down to the room temperature.

### Characterization

X-ray diffraction (XRD) and transmission electron microscopy (TEM) characterization was performed at the Institute of Functional Nano and Soft Materials (FUNSOM), Soochow University. XRD was done using a PANalytical (Empyrean) apparatus with Cu Kα as the probing source. The morphology of the as-prepared GeO_x_ was examined using TEM (Tecnai G2 F20, FEI). The Ge K-edge XAFS experiments were conducted at beamlines BL01C1 at National Synchrotron Radiation Research Center (NSRRC), Taiwan and BL12B1 at SPring8, Japan. NSRRC is a 1.5 GeV storage ring operating at the beam current of 360 mA in top-up mode. The beamline, BL01C1 has energy range of 6–33 keV and a resolution ΔE/E of 2.3 × 10^−4^. SPring-8 is an 8 GeV ring operating at the beam current of 100 mA in top-up mode. BL12B1 has an energy range of 5–25 keV with resolution ΔE/E of 10^−4^. The GeO_x_ powder was pressed into thin pellets and sealed in Kapton tape. The spectra were measured using transmission mode. Commercially obtained GeO_2_ powder (99.99%, Aladdin) was used as a reference.

### XAFS data analysis

XAFS data was processed following the standard procedure using the IFFEFIT software package^32^. Briefly, in the pre-edge region, the spectrum was fitted to a straight line, and the post-edge background was fitted with a cubic spline. The EXAFS function, χ, was obtained by subtracting the post-edge background from the overall absorption and then normalizing with respect to the edge jump step. The EXAFS fitting was performed in *R*-space between 1.0 Å and 2.6 Å (the Fourier transform from *k*-space was performed over a range of 3.0 to 14.0 Å^−1^), taking both Ge-O and Ge-Ge shells into consideration. The amplitude reduction factor S_0_^2^ was determined to be 0.85 (±0.09), using GeO_2_ powder of quartz-phase and assuming Ge is fully coordinated with a coordination number (CN) of 4. Structural parameters of the GeO_x_ samples, such as coordination numbers, bond distance (R), Debye-Waller factor (σ^2^), and inner potential shift (E_0_) were fitted using the IFEFFIT code.

## Additional Information

**How to cite this article**: Zhao, J. *et al.* Reduced GeO_2_ Nanoparticles: Electronic Structure of a Nominal GeO_x_ Complex and Its Stability under H_2_ Annealing. *Sci. Rep.* **5**, 17779; doi: 10.1038/srep17779 (2015).

## Figures and Tables

**Table 1:** EXAFS fitting parameters for GeO_x_ nanoparticles.

Name	Temperature	Shell	CN	(Å)	ΔE (eV)	σ^2^ (10^−3^ Å^2^)	R-factor
GeO2	RT	Ge-O	4	1.74(0.01)	6.33	1.4(1.0)	0.011
Fresh GeO_x_	RT	Ge-O	1.5(0.2)	1.76(0.01)	8.10	4.2(2.0)	0.006
		Ge-Ge	2.3(0.3)	2.45(0.01)	7.62	4.6(0.8)	
	300	Ge-O	1.3(0.1)	1.74(0.01)	6.88	2.9(1.1)	0.002
		Ge-Ge	2.8(0.2)	2.46(0.005)	7.74	5.4(0.5)	
	500	Ge-O	0.7(0.1)	1.73(0.01)	6.44	1.6(1.7)	0.003
		Ge-Ge	2.4(0.2)	2.46(0.005)	8.97	4.4(0.5)	
	700	Ge-O	1.1(0.2)	1.73(0.02)	7.19	3.2(2.4)	0.007
		Ge-Ge	3.5(0.8)	2.45(0.01)	6.33	4.2(1.5)	
Air-exposed GeO_x_	RT	Ge-O	3.3(0.3)	1.74(0.01)	-3.21	1.9(0.9)	0.011
		Ge-Ge	0.5(0.3)	2.48(0.01)	8.00	1.2(3.1)	
	300	Ge-O	2.5(0.2)	1.75(0.01)	-3.22	2.6(1.2)	0.004
		Ge-Ge	0.9(0.4)	2.46(0.03)	9.91	3.0(2.8)	
	500	Ge-O	2.2(0.2)	1.74(0.01)	-3.02	2.4(1.4)	0.014
		Ge-Ge	0.7(0.3)	2.46(0.02)	6.27	1.7(2.6)	
	700	Ge-O	0.2(0.1)	1.69(0.04)	-3.2	1.0(0.8)	0.010
		Ge-Ge	4.1(0.3)	2.45(0.002)	6.47	4.0(0.5)	
